# The Effect of Paddle Stroke Variables Measured by Trainesense SmartPaddle^®^ on the Velocity of the Kayak

**DOI:** 10.3390/s22030938

**Published:** 2022-01-26

**Authors:** Antti Löppönen, Tomi Vänttinen, Marko Haverinen, Vesa Linnamo

**Affiliations:** 1 Faculty of Sport and Health Sciences, University of Jyväskylä, 40014 Jyväskylä, Finland; antti.ej.lopponen@jyu.fi (A.L.); marko.haverinen@varala.fi (M.H.); 2 Physical Activity, Sports and Health Research Group, Department of Movement Sciences, KU Leuven, 3000 Leuven, Belgium; 3 KIHU-Research Institute for Olympic Sports, 40700 Jyväskylä, Finland; tomi.vanttinen@kihu.fi; 4 Varala Sports Institute, 33240 Tampere, Finland

**Keywords:** kayaking, biomechanics, canoe polo, inertial measurement unit

## Abstract

(1) Background: This study aimed to compare key variables of paddle stroke measured by a commercial Trainesense SmartPaddle^®^ against the strain-gauge shaft and investigate how these variables are associated with the velocity of the boat among national-level canoe polo players. (2) Methods: This study involved 14 Finnish national-level canoe polo players. The measurement protocol consisted of three different paddling velocities, which were performed in indoor swimming pools. The velocity of the boat was calculated based on the performance time measured with the laser photocell gate. Canoe polo equipment was used in the study and a SmartPaddle sensor was attached to the paddle blade. A strain-gauge paddle shaft was used as a reference method to examine the validity of SmartPaddle. (3) Results: The stroke rate, force production time, mean and maximal force measured with the strain-gauge paddle shaft correlated strongly (r = 0.84–0.95, *p* < 0.01) with SmartPaddle. However, the SmartPaddle overestimated the maximum force compared to the strain-gauge shaft. Stroke rate (r = 0.86, *p* < 0.01), mean force (r = 0.79, *p* < 0.01), maximal force (r = 0.78, *p* < 0.01) and total absolute impulse (r = 0.70, *p* < 0.01) correlated positively and force production time negatively (r = −0.76, *p* < 0.01) with the velocity of the boat. (4) Conclusions: We conclude that the SmartPaddle provides promising information on stroke key variables when compared to the strain-gauge paddle shaft. The SmartPaddle is a new and interesting tool for biomechanical research and daily kayaking coaching in real open water conditions. However, more research and algorithm development are needed before the SmartPaddle can be used in everyday coaching sessions in kayaking.

## 1. Introduction

To create a forward acceleration for the boat, the paddler must win the resistance forces of water and air [1,2,3,4]. Force, power, and stroke rate are the most important factors influencing the velocity of the boat [5,6,7,8,9]. In addition, it is important to produce a “bell-shaped” force profile, which leads to a bigger impulse [6,10,11]. The paddle stroke is most effective when the blade is perpendicular to the water surface [2,12] and the maximum force is reached just before this [13,14]. Studies focused on the paddle trajectory have shown that more experienced paddlers are capable of more symmetrical paddling performance [15,16,17] and have wider trajectories than inexperienced paddlers [16].

Traditionally, the direction of the paddle stroke is parallel to the kayak’s centerline, but on a wing paddle, the trajectory is slightly different because the blade’s wing shape produces buoyancy compared to a traditional paddle [11,12,15]. When the paddler is using the wing paddle, the trajectory should be on a slightly more curved line, moving beyond the centerline of the boat [15]. This has been found to prolong the optimal duration of the propulsion generation position [11]. The stroke trajectory is thus one of the key factors related to paddling performance and varies between different types of paddling such as canoe polo and sprint kayaking. Although the canoe polo is paddled with a traditional paddle (flat-blade) and should follow the same principles as other kayak sports using a traditional paddle, the actual research evidence on canoe polo is still limited.

Most of the biomechanical kayaking studies have been conducted through motion analysis and direct force measurements. Studies have been made by ergometers in laboratory conditions [8,18,19,20] and open water environments [7,10,12,21,22,23]. Studies in an open water environment often use a strain-gauge paddle shaft [6,21,23] to measure the bending of the shaft and thereby the variables of the stroke. This method has also been found to be a reliable analyze on-water rowing performance [24]. Paddle blade trajectory-related studies have previously been done with an ergometer in laboratory conditions [20,25] and open water using cinematographic cameras [12,26].

However, the limitation of these methods is that they cannot be used to study the direction of the paddle stroke or the trajectory of the paddle blade when paddling in open water conditions for long distances (competitive analyzes). Moreover, many methods such as 3D-motion analysis are not suitable for direct transfer to an open water environment especially for daily (technical) coaching sessions, where a stand-alone system is often required.

The new compact sensor technology could offer new opportunities for biomechanical research and daily coaching in water sports. The small-sized inertial measurement units allow these devices to be used in real conditions [27,28,29]. Inertial measurement units are capable of measuring, processing, and storing data in a compact size and are also very light and are thus possible to integrate into equipment [27]. Inertial measurement units have been used in water sports especially in swimming [30,31] but also in kayaking to measure kayak movements [32,33]. It is also noteworthy that the development of algorithms plays a key role as sensor technology is a growing method in the biomechanical measurement of sports. For example in swimming, stroke rate, swimming velocity, and stroke phases have been the most important variables in algorithm development [34]. However, to our knowledge, the inertial measurement units have not been used to measure similar kayaking stroke variables such as force, stroke, or force production time. The inertia measurement unit could provide a tool to estimate the direction of paddle stroke under real conditions because the data measured by its sensors can be used to determine trajectories.

In this study, we use the Trainesense SmartPaddle^®^ which is an advanced commercial wearable underwater sensor. It has been widely used in swimming to examine technique (trajectory, velocity, and orientation of the hand) and the applied forces during a swimming stroke [35]. This study aimed to compare key variables of paddle stroke measured by a SmartPaddle against the strain-gauge paddle shaft. In addition, we investigate how these key variables are associated with the velocity of the boat among national-level canoe polo players.

## 2. Materials and Methods

### 2.1. Study Design and Participants

This cross-sectional study consisted of two datasets. The first dataset (*n* = 6, all were men) compared the results of the SmartPaddle and a strain-gauge paddle shaft in key paddle stroke variables. The second study (total *n* = 14, men *n* = 12, women *n* = 2) investigated the effect of key paddle stroke variables measured on a SmartPaddle on the velocity of a boat. 

The participants of this study were Finnish national-level canoe polo players (Table 1). The average age of the dataset one participants (*n* = 6) was 39.3 ± 10.1 years, height 183.3 ± 10.6 cm, and weight 81.3 ± 13.0 kg. The average angle of the blade was 80.0 ± 0.0° and the paddle length was 206.0 ± 0.0 cm. The average age of the dataset two participants (*n* = 14) was 40.8 ± 17.2 years, height 182.3 ± 7.6 cm, and weight 81.2 ± 11.6 kg. The average angle of the blade was 69.1 ± 17.9° and the paddle length was 206.4 ± 0.9 cm.

The inclusion criterion for the participants were: (1) at least three years of experience of competition kayaking, (2) basic health, and (3) were not allowed to have any injuries or other problems that interfered with their kayaking performance. The study protocol followed the principles of the Declaration of Helsinki. The study was approved by the Human Sciences Ethics Committee of the University of Jyväskylä. All participants signed written informed consent before participating in the study. 

### 2.2. Measurement

Measurements were performed in three different indoor swimming pools, in two locations in Finland. All participants in dataset one were measured in a 25-m pool with a measurement distance of 15 m. Four participants of dataset two were measured in a 50-m pool (measuring distance 30 m) and the remaining 10 participants were measured in a 25-m pool (measuring distance 15 m). Based on the paddling velocities, the measurement distance did not affect the results of the analysis. At a 30-m analysis distance (50-m pool), mean (SD) 13.9 ± 1.7 strokes per side were included in the analysis at velocity one, 14.6 ± 2.4 strokes at velocity two, and 15.5 ± 2.1 strokes at velocity three. At a 15-m analysis distance (25-m pool), 6.4 ± 0.8 strokes were included in the analysis at velocity one, 6.9 ± 1.0 strokes at velocity two, and 7.1 ± 0.9 strokes at velocity three. The velocity of the canoe was calculated based on the performance time measured with the laser photocell gates placed on the edges of the pool. The distance between the photocells was measured and the average velocity was calculated by dividing the paddled distance by the performance time (v = s/t, where v = velocity, s = distance, and t = time). The photocells were placed in the pools so that the boat could be accelerated (acceleration distance was 5 m) to the cruising velocity before the first photocell gate started the timing.

The measurement protocol for dataset one (SmartPaddle & strain-gauge shaft comparison) consisted of three different velocities of paddling performances. First participants were instructed to paddle at a basic endurance pace (velocity 1), which was described as a slow and calm but strong pace that can be maintained for a long time. The second velocity was clearly (velocity 2) faster paddling, which was described as strong, but technically pure, paddling. This velocity was performed with three different techniques (normal, energetic beating stroke, delayed long and light stroke) to add heterogeneity to the data used in the validity analysis. The last was maximum velocity (velocity 3), which was described as a near maximum performance, where the pace is almost at maximum, but the paddling technique remains high quality. In total, dataset one contained a total of 60 data points (=6 participants × 5 different performances/participant × 2 sides (left and right). The velocities one (1.95 ± 0.22 m/s) two (2.20 ± 0.21 m/s) and three (2.50 ± 0.20 m/s) differed significantly (*p* < 0.05) as planned. The velocities of the two different technical styles (energetic beating stroke 2.36 ± 0.26 m/s & delayed long and light stroke 2.25 ± 0.21 m/s) were the same as normal style of velocity two (*p* = 0.313, *p* = 0.563. respectively) as planned.

Dataset two consisted of the three different velocities with normal paddling performances mentioned above (velocity 1, 2 & 3), to study the effect of key paddle stroke variables measured on a SmartPaddle on the velocity of a boat. Dataset two contained a total of 84 data points (=14 participants × 3 different velocity performances/participant × 2 sides (left and right)). The velocity of the boat differed significantly between different velocities (*p* < 0.001) as planned.

### 2.3. Instrumentation

**Figure 1:**
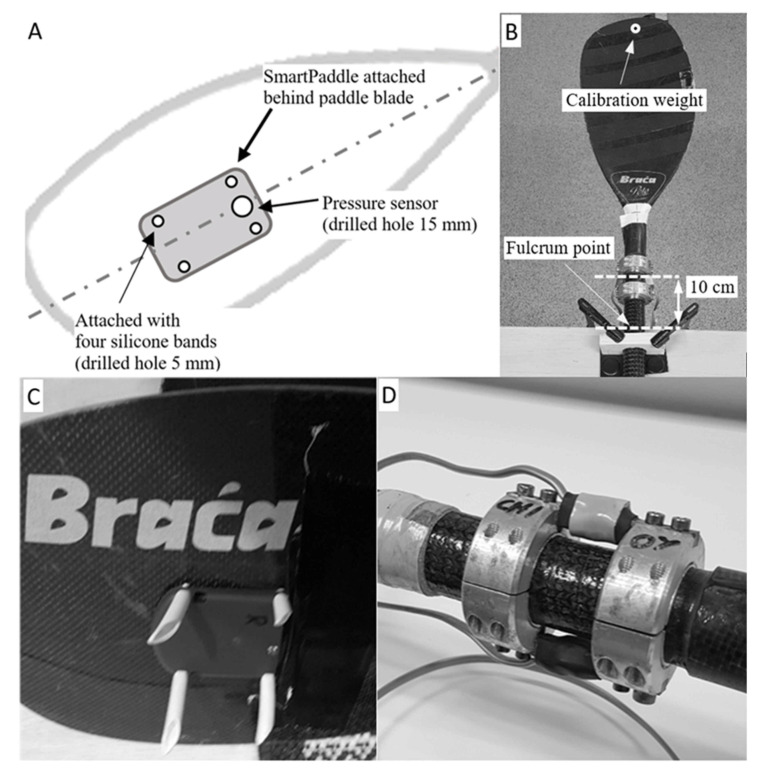
(**A**) SmartPaddle place on the blade, (**B**) Strain-gauge shaft in calibration board (without IMU attached), (**C**) SmartPaddle attached to the blade, (**D**) structure of the strain-gauge sensors.

The commercial SmartPaddle (Trainesense Oy, Tampere, Finland) was used as a measuring unit (9-axis IMU + pressure sensor). The device uses a sampling frequency of 100 Hz and the data was stored in the Trainesense Analysis Center cloud service. The data was uploaded to the mobile phone and onward Analysis Center via a Bluetooth connection. The SmartPaddle was mounted on the back of the blade (Braca-Sport Polo Kinetic max, adjustable shaft, zero-length 205 cm) using silicone straps, and a 15 mm diameter hole was drilled in the paddle blade for the pressure sensor (Figure 1).

A strain-gauge paddle shaft (self-manufactured, University of Jyväskylä, Finland) [9] was used for the comparison of the SmartPaddle (Figure 1D). This same strain-gauge equipment has also been used in previous research when examining the relationships between force-time parameters and kayak velocity [9]. The data produced by the strain-gauge sensors were amplified with separate amplifiers (TRtesti Oy, Jyväskylä Finland) and stored on a recordable AD converter (self-manufactured “LIIKE card”, Sports Technology, University of Jyväskylä, Vuokatti, Finland) at a frequency of 250 Hz. The gauge paddle shaft was calibrated using 0 kg, 4 kg, and 10 kg weights to calculate the linear gain factor. During the calibration process, the strain gauge shaft was attached to the calibration table with the clamps and the fulcrum point was at the edge of the table (Figure 1B). The linear gain factor determined from the calibration was entered into the recordable AD Converter analysis software. 

### 2.4. Data Processing

The SmartPaddle calculation algorithm has not been published. The SmartPaddle generates the processed data via the closed Matlab GUI (Graphical User Interface) (Tampere, Finland) developed by the Trainesense Oy (Tampere, Finland) meaning that there is no access to SmartPaddle raw data and the algorithm calculation constants can not be adjusted. This was also the reason why the comparisons between the strain-gauge shaft and the SmartPaddle used averaged variables as it was not possible to perform the actual stroke (SmartPaddle)-stroke (strain-gauge shaft) synchronization due to lack of the raw data from the SmartPaddle. In the present study, the SmartPaddle variables were normalized to paddle blade (Bracsa polo kinetic max, 735 cm^2^) area and the data were processed separately for the right and left blades. 

The mean force was the average total force for that time when the blade is in the water. The maximum force was the highest total (all directions) force that the SmartPaddle registered during the entire performance. The force production time was defined as the duration during which the blade produced force at more than 30% of the stroke maximum force. The absolute total impulse was total force multiplied by the whole time when the blade was under the water surface [35]. The SmartPaddle analysis consisted of only the cruising phase of the performances without the acceleration and braking phases. All variables except maximum force were averages of the strokes included in the analysis. The relative forward, lateral and vertical impulses estimated by the SmartPaddle were calculated in line with kayaking direction.

The data stored from the strain-gauge paddle shaft was processed using Matlab (R2020b, The MathWorks Inc., Natick, MA, USA). First, the raw data were filtered using a moving-average filter (movmean function) with a value of 10 data points (filter window) [36], to attenuate the largest interference peaks. Then the maximum force (max–function) of the paddle stroke was determined from the filtered data by identifying a peak force value (highest force) of the entire performance (cruising phase). Finally, mean stroke force and force production time were calculated using the same 30% force limit as the SmartPaddle algorithm in order to make the analysis comparable. This was done by determining a level of 30% of the maximum force. The duration and mean of the force production time were determined from the force vector above this level. The left and right sides were processed separately in the analysis, with both sides forming their own data points in the statistical analysis. Mean values (except maximum force) of total kayaking performances (cruising phase) were used in this strain-gauge paddle shaft analysis to make it comparable with the SmartPaddle analysis.

### 2.5. Statistical Analysis

Results are reported as mean and standard deviation (SD). Shapiro-Wilk normality test and histograms visual evaluation (normal distribution curve and distribution skewness and kurtosis) were used to check the normality of the data. The Shapiro-Wilk test indicated that some of the variables were not normally distributed (stroke rate, boat velocity, lateral and vertical relative impulse). Similarly, the visual interpretation of the histograms and the small sample size (*n* = 6 & *n* = 14) supported the selection of non-parametric tests. Associations between the data measured by the SmartPaddle and the strain-gauge paddle shaft were tested with the Spearman correlation coefficient. The correspondence between the data measured by the SmartPaddle and the gauge-paddle shaft was evaluated with two-way random (absolute agreement), single measure intra-class correlation coefficients (ICC) [37], and visualized with Bland-Altman plots [38]. ICC was used to characterize the correspondence as poor (<0.40), fair (0.40 to <0.60), good (0.60 to <0.75), or excellent (≥0.75) [39]. The relationship between velocity and key paddle variables measured with the SmartPaddle was also studied by the Spearman correlation coefficients (two-tailed). Differences between velocities groups and strain-gauge paddle shaft and the SmartPaddle were tested using related–samples Wilcoxon signed-rank test. Statistical significance was set at *p* ≤ 0.05 (two-tailed) and analyses were performed with the R statistical computing software (version 4.0.3) [40]. 

## 3. Results

### 3.1. Strain-Gauge Shaft Comparison

The SmartPaddle measured higher maximum force values than the strain-gauge paddle shaft (*p* < 0.001) (Table 2). The strain-gauge paddle shaft maximum force was 125.4 ± 34.2 N, while the mean of the maximum force of the SmartPaddle was 152.1 ± 57.4 N. However, the correlation between SmartPaddle and strain-gauge paddle shaft was strong 0.84 (*p* < 0.001), and the ICC was classified to be good 0.64 (Confidence Interval CI 95% 0.21, 0.82) (*p* = 0.003). The strain-gauge paddle shaft mean force was 85.4 ± 25.3 N and the SmartPaddle 83.3 ± 30.3 N, with a strong correlation of 0.88 (*p* < 0.001) and an excellent ICC of 0.86 (CI, 0.78, 0.92) (*p* < 0.001). This difference in mean force was not statistically significant between these two methods (*p* = 0.083). 

**Figure 2:**
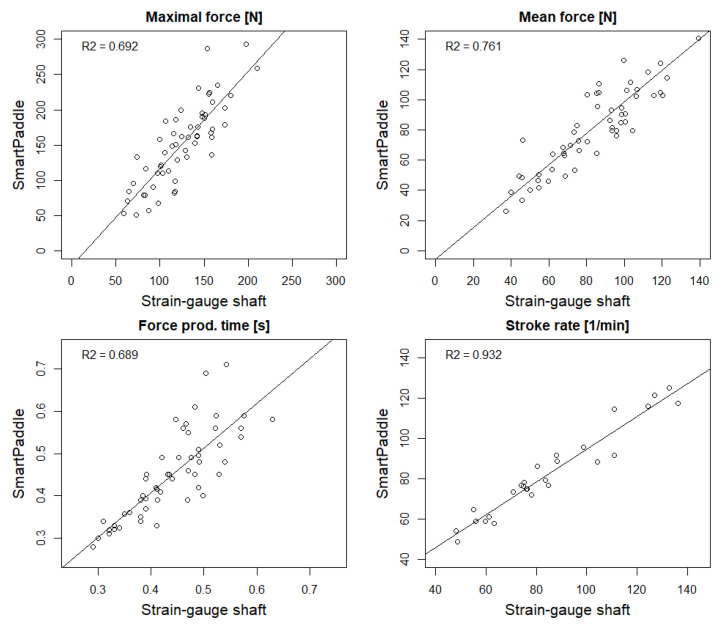
Scatter plots and R^2^ values between SmartPaddle and strain-gauge shaft in key paddle stroke variables (*n* = 6) (R^2^ = coefficient of determination showing how close the data are to the fitted regression line).

The force production time strongly correlated between the strain-gauge paddle shaft and the SmartPaddle (0.88, *p* < 0.01) and the average difference was only 0.01 s (*p* = 0.472) The ICC was classified to be excellent 0.81 (CI, 0.70, 0.88) (*p* < 0.001). Of all the variables, the strongest correlation was found in the stroke rate 0.95 (*p* < 0.01) and it did not differ statistically (*p* = 0.199), and the ICC was excellent at 0.95 (CI, 0.89, 0.98) (*p* < 0.001) (Table 2). Scatter plots and R^2^ values between SmartPaddle and strain-gauge shaft in key paddle stroke variables are presented in Figure 2.

**Figure 3:**
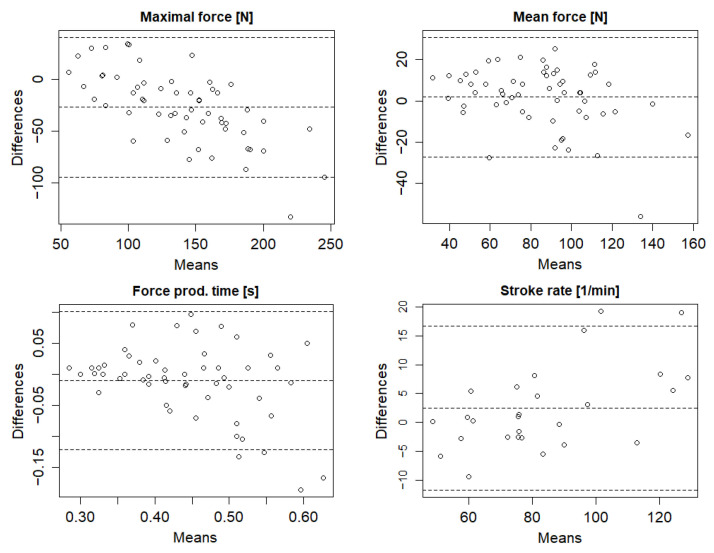
Bland–Altman analysis between SmartPaddle and strain-gauge shaft in key paddle stroke variables (*n* = 6).

The Bland-Altman analysis (Figure 3) showed that there were only a few cases outside the concordance limits (95%) and no systematic error difference was noticeable, as the measurement results were evenly distributed on both sides of the zero level.

### 3.2. Key Variables to Velocity

Stroke rate (r = 0.86, *p* < 0.001) and mean force (r = 0.79, *p* < 0.001) were most strongly related to boat velocity and differed between all kayaking velocities (*p* < 0.001) (Table 3 and Table 4). The absolute impulse (r = 0.70, *p* < 0.001) was also positively related to the velocity of the boat and differed significantly (*p* < 0.001) between the velocities while the force production-time was negatively related (r = −0.76, *p* < 0.001) to the velocity of the boat.

The relative lateral impulse was positively and moderately related to boat velocity (r = 0.42, *p* < 0.001) while the vertical impulse was negatively and moderately related to velocity (r = −0.42, *p* < 0.001). There was no change in the forward impulse as the velocity increased and it did not correlate significantly with the velocity (r = −0.09, *p* = 0.429).

## 4. Discussion

The present study suggests that the Trainesense SmartPaddle^®^ offers promising opportunities to measure paddle stroke key variables acknowledging that the calculation algorithms and device mounting need to be further developed more suitable for kayaking in the future. The stroke rate, force, force-time, and total absolute impulse were found to be key variables affecting the velocity of the boat among canoe polo players and these results were also well in line with previous studies with sprint kayakers [5,8]. Moreover, the Trainesense SmartPaddle^®^ made it possible to study the direction of the stroke, and in this study, when the velocity of the boat increased, the relative amount of the lateral impulse of the paddle stroke increased, while the vertical decreased. The Trainesense SmartPaddle can provide new insights for technical coaching in kayaking in real open water conditions.

In this study, good to excellent agreement was found between the SmartPaddle and the strain-gauge shaft in key variables of paddle stroke (0.64–0.95, *p* < 0.001). The absolute differences between the SmartPaddle and the reference method in force production time, mean force, and stroke rate were small. The SmartPaddle overestimated maximum force compared to a strain-gauge shaft (*p* > 0.001) and the agreement was also lowest in this variable (0.64, *p* < 0.001). This may be due to differences in data processing, especially data filtering. We used a moving average (movmean) filter to analyze the strain-gauge shaft data, which may cut the highest force peaks more than the filtering algorithm used by the SmartPaddle. However, it was more likely that the SmartPaddle overestimated the highest values such as maximum force and absolute impulse due to issues related to their hardware and algorithms. This was indicated by the sensitivity analysis between the left and right sides which suggested that it was indeed the SmartPaddle that overestimated the maximum force compared to the strain-gauge shaft (Supplementary Table S1). Unfortunately, this difference could not be studied further because the algorithm used by the SmartPaddle is a trade secret and therefore these estimates should be treated with caution.

To our knowledge, inertial measurement units have not previously been used to measure the trajectory of the paddle in a real open water environment. Furthermore, to our knowledge, this is the first study to measure kayak paddling technique under real conditions through a paddle attached IMU sensor. The advantage of SmartPaddle is that it can be used to identify the direction of the paddle stroke. The results of this study suggested that as the velocity of the boat increased, the relative amount of lateral impulse of the paddle stroke increased, vertical decreased, and no difference was observed in the relative amount of forward impulse. This observation suggests that when boat velocity increases, paddles tend to produce more force by changing the trajectory of the paddle blade. On the other hand, this may be affected by the fact that the stroke is shorter at a higher stroke rate [6,10] and thus the vertical impulse is lower. This observation has been found in the previous studies which have shown that as the stroke rate becomes sufficiently high, the duration of the water phase also shortens, especially from the beginning phase of the stroke (first 1/3) and the end phase (last 1/3) of the stroke [21]. However, it should be noted that this study used a standard blade (canoe polo blade) which has been found to differ from the wing paddle trajectory [15]. The results of the left and right sides comparison indicated (Supplementary Table S2) that there was a slight difference in the direction of the strokes between the sides which is in agreement with the previous studies in which asymmetries between left and right strokes trajectories [6,20,33,41].

Although the results provide indications that the SmartPaddle, which is widely used in swimming, can be used to measure the biomechanics of kayaking, more studies on sensor reliability and validity are still needed. Furthermore, the SmartPaddle appears to work well on key variables of paddle stroke based on these results, but the trajectory or direction of the paddle blade has not been validated in this or previous studies. This needs to be done in the future, especially if SmartPaddle is used in research. It is especially important to study how the sensor can measure variables on the wing paddle as one possible limitation may be the turbulent vortex [11,42] in the blade surface. In addition, further research is needed to find out why the SmartPaddle seemed to overestimate the maximum force compared to the strain-gauge shaft.

It has previously been found that there is a strong correlation between the average velocity of the kayak and the total impulse, regardless of the frequency, which would suggest that only by increasing the total impulse can the velocity of the kayak be increased [10]. In the future, it would be valuable to study the relationship between the forward impulse and the stroke rate to the kayak velocity. This could serve as one of the important efficiency indicators in kayaking, because this analysis makes it possible to estimate the proportion of the forward impulse.

Some limitations need to be kept in mind when interpreting the findings. Generalization of the results is restricted, particularly for sprint and marathon kayaking, as they exclusively use wing blades and the measurements in this study were performed on canoe polo equipment. In addition, the subjects’ average age in this study was high (40.8 years) and standard deviation large (±17.2 years), which also impairs generalization of the results for younger international level athletes. It should be noted that the distances in this study were shorter than in the actual exercises, and for successful use in them SmartPaddle attachment to the paddle blade must be developed in the future and possibly integrated into the structure of the blade.

The strength of this study was that the measurement protocol was successful, the velocities were significantly different as was planned, and the paddling technique was pure. Conditions were standardized in the swimming pools so that pool depth, water temperature, or weather conditions did not affect the results. This study focused on the cruising velocities, as it is known from previous research [43] that the first accelerator paddle strokes differ from the cruising velocity paddle strokes and thus the data processing was more reliable. Participants in the study were experienced kayakers and their paddling techniques were well established. Thus, the performances were clean, and their technique was stable throughout the performance, although it could not be fully standardized due to individual differences. In this study, subjects did not report problems even though the device was attached to the paddle blade.

The SmartPaddle could support traditional video coaching in kayaking and strengthening the vision of coaches and athletes. If the development of the algorithm is continued, it should be done according to the requirements of kayaking, so that the device can operate reliably in training sessions and competition analyses to evaluate kayak paddling techniques in all types of kayak sports.

## 5. Conclusions

The SmartPaddle, consisting of a 9-axis IMU and pressure sensor, offers promising opportunities to measure stroke key variables when compared to the strain-gauge paddle shaft. Stroke rate, force, force production time, and total impulse were the stroke key variables, which affect the velocity of the kayak. The results of the study give preliminary indications that when the velocity of the kayak increases, the relative amount of lateral impulse increases, and the vertical impulse decreases while the forward impulse remains the same. Although kayaking has been extensively studied using motion analysis and force sensors, the study of the direction of paddle stroke has been done less. The results presented in this study suggest that the SmartPaddle can be attached to the paddle blade and thus provide a new way to study the direction of the paddle stroke in real open water conditions. However, more research and algorithm development are needed, especially to determine the reliability of the trajectory of the blade and the direction of stroke.

## Figures and Tables

**Table 1:** The descriptive information of the study participants.

Mean (SD)	1st Dataset (*n* = 6) ^1^	2nd Dataset (*n* = 14) ^1^
Age [years]	39.3 ± 10.1	40.8 ± 17.2
Height [cm]	183.3 ± 10.6	182.3 ± 7.6
Weight [kg]	81.3 ± 12.9	81.2 ± 11.6
Blade angle [deg]	80.0 ± 0.0 ^2^	69.1 ± 17.9
Paddle length [cm]	206.0 ± 0.0 ^2^	206.4 ± 0.9

SD = Standard Deviation, ^1^ Descriptive table is missing data from five participants because they did not reply to the questionnaire sent afterward (1st dataset = 2 participants, 2nd dataset = 3 participants). ^2^ no adjustable.

**Table 2:** Comparison between the strain-gauge paddle shaft and the SmartPaddle (9-axis IMU+ pressure sensor) in key paddle stroke variables (*n* = 6).

Mean (SD)	Strain-Gauge	SmartPaddle	*p*-Value ^1^	r ^2^	ICC ^3^
Maximal force [N]	125.4 ± 34.2	152.1 ± 57.4	<0.001	0.84 **	0.64 ***
Mean force [N]	85.4 ± 25.3	83.3 ± 30.3	=0.083	0.88 **	0.86 ***
Force prod. time [s]	0.43 ± 0.08	0.44 ± 0.10	=0.472	0.88 **	0.81 ***
Stroke rate [1/min]	84.8 ± 25.6	80.7 ± 21.0	=0.199	0.95 **	0.95 ***

SD = Standard deviation, ^1^ Wilcoxon signed rank test, ^2^ Spearman’s rank correlation coefficient, ^3^ ICC = Intraclass Correlation Coefficient, ** Correlation is statistically significant 0.01 level (2-tailed), *** *p* < 0.001.

**Table 3:** Descriptive characteristics of the participants and results of the testing velocities (*n* = 14).

Mean (SD)	Velocity 1	Velocity 2	Dif V1–V2 ^1^	Velocity 3	Dif V2–V3 ^1^
Velocity [m/s]	2.1 ± 0.2	2.4 ± 0.1	<0.001	2.7 ± 0.3	<0.001
Maximal force [N]	112.6 ± 38.4	145.1 ± 40.1	<0.001	224.9 ± 67.8	<0.001
Mean force [N]	61.6 ± 17.9	83.6 ± 21.8	<0.001	115.8 ± 32.2	<0.001
Force prod. time [s]	0.46 ± 0.06	0.40 ± 0.06	<0.001	0.34 ± 0.07	<0.001
Stroke rate [1/min]	75.7 ± 14.3	93.5 ± 13.4	<0.001	119.3 ± 19.7	<0.001
Total impulse abs [Ns]	58.6 ± 16.8	69.2 ± 15.8	<0.001	90.1 ± 22.8	<0.001
Impulse forward [%] ^2^	49.50 ± 4.29	50.36 ± 4.23	0.387	49.75 ± 4.05	0.770
Impulse lateral [%] ^2^	28.81 ± 4.90	29.60 ± 5.10	0.058	32.08 ± 5.14	0.036
Impulse vertical [%] ^2^	21.69 ± 4.45	20.04 ± 3.81	0.002	18.17 ± 4.24	0.009

SD = Standard deviation, ^1^ Related-samples Wilcoxon signed-rank test, ^2^ Portion of total impulse.

**Table 4:** The Spearman’s rank correlation coefficient by velocity groups (*n* = 14).

	Velocity 1	Velocity 2	Velocity 3	All Velocity
Maximal force [N]	0.60 **	0.56 **	0.74 **	0.78 **
Mean force [N]	0.61 **	0.47 *	0.73 **	0.79 **
Force prod. time [s]	−0.54 **	−0.40 *	−0.56 **	−0.76 **
Stroke rate [1/min]	0.82 **	0.34	0.76 **	0.86 **
Total impulse abs [Ns]	0.43 *	0.52 **	0.74 **	0.70 **
Impulse forward [%]	−0.15	−0.01	−0.18	−0.09
Impulse lateral [%]	0.45 *	0.11	0.58 **	0.42 **
Impulse vertical [%]	−0.27	−0.07	−0.46 *	−0.42 **

* *p* < 0.05, ** *p* < 0.01 (two-tailed).

## Data Availability

Pseudonymized datasets are available to external collaborators subject to agreement on the terms of data use and publication of results. To request the data, please contact Antti Löppönen (antti.ej.lopponen@jyu.fi).

## Supplementary Materials

The following are available online at https://www.mdpi.com/article/10.3390/s22030938/s1, Table S1: Left- and right-side comparison between strain-gauge shaft and SmartPaddle; Table S2: Left- and right-side comparison between velocities.
