# Supplementary material for: The Effect of Paddle Stroke Variables Measured by Trainesense SmartPaddle® on the Velocity of the Kayak
Source: Sensors (Basel). 2022 Jan 26;22(3):938. doi: 10.3390/s22030938 (PMC8840261; doi:10.3390/s22030938)
Supplement: Supplementary file 1 [file sensors-22-00938-s001.zip › sensors-1480804-supplementary.pdf]

Table S1. Left- and right-side comparison (mean (SD)) between strain-gauge shaft and SmartPaddle (n = 6).

		Left	Right	Total
Maximal force [N]	Strain-gauge shaft	139.3 (35.4)	111.6 (27.1)	125.4 (34.2)
	SmartPaddle	164.7 (57.5)	139.9 (55.6)	152.1 (57.4)
	p-value[a]	.001	< .001	
	Spearman's correlation	0.79 **	0.88 **	
Force prod. time [s]	Strain-gauge shaft	0.44 (0.09)	0.43 (0.08)	0.43 (0.08)
	SmartPaddle	0.45 (0.10)	0.44 (0.10)	0.44 (0.10)
	p-value[a]	.749	.471	
	Spearman's correlation	0.87 **	0.90 **	
Mean force [N]	Strain-gauge shaft	94.1 (27.0)	76.7 (20.3)	85.4 (25.3)
	SmartPaddle	88.2 (30.3)	78.6 (30.0)	83.3 (30.3)
	p-value[a]	.027	.804	
	Spearman's correlation	0.89 **	0.87 **	

[a] Related-Samples Wilcoxon Signed Rank Test, ** Correlation is significant at the 0.01 level (2-tailed).

Table S2. Left- and right-side comparison between velocities (n = 14).

	Velocity 1					Velocity 2					Velocity 3				
	left		right			left		right			left		right		
	Mean	SD	Mean	SD	p-value[a]	Mean	SD	Mean	SD	p-value[a]	Mean	SD	Mean	SD	p-value[a]
Maximal force [N]	128.5	40.3	96.7	29.8	**.004**	156.7	41.3	133.5	36.6	**.022**	231.1	67.7	218.7	70.0	.695
Mean force [N]	63,6	17,1	59,7	19,0	.363	85,0	20,6	82,2	23,6	.638	115,2	31,2	116,5	34,5	.075
Force prod. time [s]	0,46	0,06	0,46	0,07	.582	0,41	0,05	0,40	0,07	.271	0,34	0,06	0,33	0,08	.306
Stroke rate [1/min]	38,5	6,6	37,2	7,9	.091	46,9	6,9	46,6	6,8	.382	60,6	8,7	58,6	11,2	.575
Total impulse abs [Ns]	64,4	16,7	52,8	15,3	**.022**	73,1	15,6	65,2	15,5	.064	94,1	22,6	86,1	23,2	.814
Impulse forward [%]	47,4	4,3	51,5	3,4	**.033**	49,6	5,0	51,1	3,3	.594	48,0	3,5	51,5	3,9	**.005**
Impulse lateral [%]	28,0	6,3	29,5	3,2	.422	28,7	5,4	30,5	4,8	.124	31,8	5,8	32,3	4,6	.937
Impulse vertical [%]	24,6	4.4	19,0	2,4	**.002**	21,7	4,3	18,4	2,5	**.016**	20,2	4,9	16,2	2,2	**.005**

N = Newton, Ns = newton-second, [a] Related-Samples Wilcoxon Signed Rank Test
